# Proof-of-Concept for Long-Term Human Endometrial Epithelial Organoids in Modeling Menstrual Cycle Responses

**DOI:** 10.3390/cells13211811

**Published:** 2024-11-02

**Authors:** Yanyu Jiang, Arturo Reyes Palomares, Patricia Munoz, Ivan Nalvarte, Ganesh Acharya, Jose Inzunza, Mukesh Varshney, Kenny Alexandra Rodriguez-Wallberg

**Affiliations:** 1 Laboratory of Translational Fertility Preservation, Department of Oncology and Pathology, Karolinska Institutet, 171 76 Stockholm, Sweden; yanyu.jiang@ki.se (Y.J.); arturo.reyes.palomares@ki.se (A.R.P.); 2 Department of Biosciences and Nutrition, Karolinska Institute, 141 52 Huddinge, Sweden; patricia.munoz.salvatierra@ki.se (P.M.); ivan.nalvarte@ki.se (I.N.); jose.inzunza@ki.se (J.I.); 3 Department of Neurobiology, Care Sciences and Society, Division of Neurogeriatrics, Karolinska Institute, 171 76 Stockholm, Sweden; 4 Department of Clinical Science, Intervention and Technology-CLINTEC, Karolinska Institute, 141 52 Huddinge, Sweden; ganesh.acharya@ki.se; 5 Center for Fetal Medicine, Karolinska University Hospital, 171 76 Stockholm, Sweden; 6 Department of Laboratory Medicine, Karolinska Institute, 141 52 Huddinge, Sweden; 7 Department of Reproductive Medicine, Division of Gynecology and Reproduction, Karolinska University Hospital, 171 76 Stockholm, Sweden

**Keywords:** endometrium growth, sequential hormone treatment, organoid culture, proliferative phase, secretory phase, estrogen, progesterone

## Abstract

Endometrial disorders, such as infertility and endometriosis, significantly impact reproductive health, thus necessitating better models to study endometrial function. Current in vitro models fail to replicate the complexity of the human endometrium throughout the entire menstrual cycle. This study aimed to assess the physiological response of human endometrial organoids (hEOs) to in vitro hormonal treatments designed to mimic the hormonal fluctuations of the menstrual cycle. Endometrial biopsies from three healthy women were used to develop hEOs, which were treated over 28 days with three hormonal stimulation strategies: (1) estrogen only (E) to mimic the proliferative phase, (2) the addition of progesterone (EP) to simulate the secretory phase, and (3) the further addition of cAMP (EPC) to enhance the secretory functions of hEOs. Gene and protein expression were analyzed using qPCR, IHC, and ELISA. The hEOs exhibited proliferation, gland formation, and appropriate expression of markers such as E-cadherin and Ki67. The hormonal treatments induced significant changes in *PR*, *HSD17B1*, *PAEP*, *SPP1*, and other genes relevant to endometrial function, closely mirroring in vivo physiological responses. The prominent changes were observed in EPC-treated hEOs (week 4) with significantly high expression of uterine milk components such as glycodelin (PAEP) and osteopontin (SPP1), reflecting mid- to late-secretory phase physiology. This model successfully recapitulates human menstrual cycle dynamics and offers a promising platform for studying endometrial disorders and advancing personalized treatments in gynecology.

## 1. Introduction

The human endometrium, a dynamic tissue essential for reproduction through embryo implantation and placental development, presents unique challenges in disease modeling and pathophysiology due to its rapid cyclical transformation and constant regeneration [1,2]. Moreover, endometrium injuries caused by cancer treatment using radiation therapy or curettages indicated by benign conditions can result in uterine factor infertility in women [3]. While technologies for protecting fertility potential currently include the use of assisted reproductive technologies and cryopreservation methods applied to gametes or embryos [4], methods of protecting and repairing injured endometrium are lacking. Endometrial regeneration has been proposed using heterologous menstrual blood-derived stem cells, bone marrow-derived mesenchymal stem cells, endometrial stem cells, and umbilical cord-derived stem cells [5,6,7]. However, the responsiveness of such cells to hormone exposure in vitro, mimicking the human menstrual cycle, is not clear yet. The advancements in organoid and three-dimensional (3D) culture techniques have opened new avenues for endometrial studies, with organoids closely depicting the tissue of origin in both structure and function [8]. These models do not completely simulate the full complexity of the system, as they lack essential components necessary for mimicking pathophysiology, such as blood and immune systems and clotting mechanisms. Microfluidic systems have been explored for integrated in vitro culture strategies to mimic physiological tissue–tissue interactions, such as between endometrium and ovarian tissue [9]. However, their reliance on a combination of human and animal tissues may limit their relevance to biology. Thus, further research is needed to better profile individual hormonal responses in vitro. The utility of endometrial organoids, particularly those derived from biopsies, holds promise in modeling diseases and facilitating potential therapeutic strategies in regenerative medicine [10]. Endometrial organoids have been used to model uterine diseases [11,12], but they have not been used yet for endometrium regeneration. Recent research indicates that endometrial organoids can respond to 2 days of estrogen and 4 days of progesterone treatment in culture [13]; however, it is not known if endometrium organoids can functionally recapitulate a full menstrual cycle. The focus of this study was to assess the human biopsy-derived endometrial organoids (hEOs) as viable in vitro models that can mimic the full in vivo menstrual hormonal cycle. To delineate this, a sequential hormonal treatment was administered to hEOs, imitating proliferative and secretory phases of the menstrual cycle over 28 days, with responsiveness gauged through biomarkers specific to each phase. The menstrual cycle is characterized by two key phases, each regulated by distinct hormonal influences. During the proliferative phase, driven by estrogen, the endometrium thickens, and the glandular epithelium proliferates. Estrogen receptor alpha (ESR1), a key mediator of estrogen signaling, rises during this phase, promoting endometrial receptivity, while the overexpression of ESR1 can lead to pathologies like endometrial hyperplasia [14]. Concurrently, Ki67, a marker of cellular proliferation, increases, reflecting the active cell division in the epithelium [15]. As the cycle progresses to the secretory phase, progesterone becomes the dominant hormone, facilitating the transition to a receptive endometrium [16]. Progesterone receptor (PR) and 17β-hydroxysteroid dehydrogenase (HSD17B1), both regulated by progesterone, are critical during this phase. HSD17B1, which mediates local estrogen metabolism, is particularly significant in endometrial epithelial cells, with its expression peaking during the late secretory phase [17,18]. Additionally, markers such as Forkhead box protein O1 (FOXO1), which regulates cellular differentiation, and progesterone-associated endometrial protein (PAEP) (glycodelin), essential for immune modulation and embryo implantation, are upregulated by progesterone [19,20]. Other key molecular players, such as secreted phosphoprotein 1 (SPP1), mucin 1 (MUC1), and leukemia inhibitory factor (LIF), further contribute to creating an environment conducive to embryo attachment and implantation. These markers underscore the importance of hormonal regulation in achieving endometrial receptivity [21,22]. Furthermore, cyclic adenosine monophosphate (cAMP) acts synergistically with progesterone to enhance the secretory function of glandular epithelial cells. This interaction promotes the production of glycodelin (PAEP) and osteopontin (SPP1), both of which are crucial for embryo attachment [22]. By evaluating the combined effects of cAMP and progesterone on glandular epithelial cells, our study aims to replicate the hormonal dynamics of the menstrual cycle in vitro, providing a comprehensive model for studying endometrial function. Through the integration of these molecular markers into our human endometrial epithelial organoid (hEO) model, we aim to gain a deeper understanding of endometrial remodeling across the menstrual cycle. This model holds great potential for studying reproductive disorders and exploring therapeutic strategies, such as regenerative medicine and endometrial transplantation.

## 2. Materials and Methods

### 2.1. Endometrial Samples

**Figure 1:**
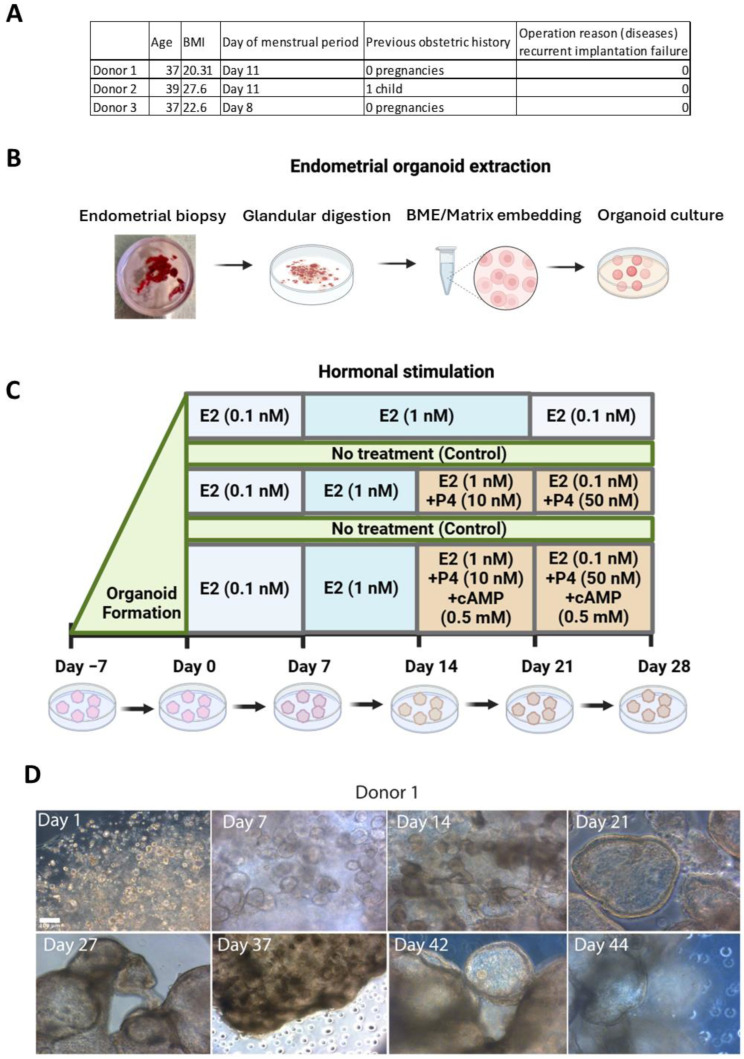
Endometrial organoids derived from donor biopsy and maintained as glandular structures in long-term culture: (**A**) anonymized clinical data of donors; (**B**) extraction of glandular structures and organoid culture establishment; (**C**) hormone treatment conditions of endometrial organoids for 28 days period; (**D**) representative bright field images of endometrial organoids from Donor 1 at different days in long-term culture in spinning bioreactor. Scale bar 100 µm for image panel in (**D**).

Endometrium biopsies from three women of reproductive age, with regular menstrual cycles and no known endometrial abnormalities, were collected using Pipelle^®^ (endometrial suction curette, Cooper Surgical, Trumbull, CT, USA) in the proliferative phase of the menstrual cycle (d7–d14) (Figure 1A) at the Reproductive Medicine Clinic of Karolinska University Hospital. None of the donors were using hormonal contraception at the time of collection. The biopsies were obtained during routine clinical visits, where the opportunity arose to collect endometrial samples. Ethical approval (reference number: 2021-06299-01) was obtained from the Swedish Ethical Review Authority. Written informed consent was obtained from all participants.

### 2.2. Human Endometrial Organoid Establishment and Culture

On receipt, tissue was collected in a 20 mL tissue collection medium (Supplementary Table S1) on ice and processed within one hour. Tissue was carefully tipped into a 100 mm Petri dish, followed by mincing with a sterile scalpel to 0.5–1 mm^3^ pieces, and digested with enzymatic solution (Supplementary Table S1) for 40 min with constant shaking on an orbital shaker at 37 °C. The tissue digest was filtered through 100 µm cell strainers (Miltenyi Biotec, Bergisch Gladbach, Germany) to retrieve glands while passing the stromal cells. Glandular structures were resuspended in 600 ul ice-cold human endometrial media (hEOM) (Supplementary Table S2) and 1.4 mL basement membrane extract (BME/Geltrex) (ThermoFisher Scientific, Waltham, MA, USA) while being kept on ice. For long-term culture, domes of glandular suspension in 20 uL volume were dispensed in 100 mm Petri dishes and incubated for 30 min in the incubator (Figure 1B), followed by the addition of 15 mL of a prewarmed hEOM medium. For hormonal treatment, domes were plated in 12-well plates (3 droplets per well), followed by each treatment performed in triplicate for each donor. Additionally, a fourth well for each treatment was kept for histology purposes. Glandular structures were cultured in a CO_2_ incubator at 37 °C with medium change every 2–3 days. Domes for long-term culture (up to 44 days) were gently flushed with the media in a Petri dish and transferred (8 domes/well) to a 12-well plate-spinning bioreactor as described previously [23], while the rest were used for hormone treatment after 7 days of culture.

### 2.3. Hormone Treatment

hEOs were divided into subgroups according to the hormone treatment regimen presented in Figure 1C. Doses of hormones used were as follows: 0.1 nM (day 0–7), 1 nM (day 7–21) and 0.1 nM (day 21–28) for estrogen (E2) (Merck, Darmstadt, Germany); 10 nM (day 14–21) and 50 nM (day 21–28) for progesterone (P4) (Merck, Darmstadt, Germany); and 0.5 mM dibutyryl cyclic adenosine monophosphate (AMP) (dbcAMP, day 14–21 and day 21–28) (MedChemExpress, South Brunswick, NJ, USA). Control (untreated) hEOs were included for every 7 days of the corresponding hormone treatment regimen until day 28. hEOs were collected for analysis every week from their respective treatment group (Figure 1C).

### 2.4. Real-Time Quantitative Polymerase Chain Reaction (RT-qPCR)

Organoid domes from each treatment well were collected in nuclease-free microfuge tubes and briefly treated with ice-cold DPBS for 10 min to release the matrix (BME), followed by supernatant removal and the addition of 350 µL of RLT buffer (Qiagen, Hilden, Germany); then, they were snap-frozen and stored at −20 °C until use. Total RNA was extracted with an RNeasy Mini Kit (Qiagen, Hilden, Germany) and cDNA was synthesized with a SuperScript™IV VILO™ Master Mix Kit (Invitrogen, Waltham, MA, USA) according to manufacturers’ instructions. The obtained cDNA was utilized for qPCR reactions using KAPA FAST SYBR MIX and target-specific primers (Supplementary Table S3) on an ABI 7500 FAST thermal cycler (Applied Biosystems, Waltham, MA, USA). GAPDH and HPRT1 were used as housekeeping genes.

### 2.5. Tissue Preparation and Immunofluorescence (IF) Staining

Following respective treatments, organoids were collected in 1.5 microfuge tubes, the excess medium was removed, and 1 mL of ice-cold 4% paraformaldehyde (PFA) was dispensed, followed by fixation for 30 min at RT. PFA was removed carefully, followed by 2 washes with PBS 5 min each. Fixed organoids were cryoprotected in 30% sucrose at 4 °C overnight, followed by embedding in OCT compound and sectioned at 7–14 µm in a cryotome. Sections were washed twice in PBS–glycine for 10 min each and permeabilized with 0.3% Triton-x-100, blocked with 10% normal goat serum (Thermo Fisher Scientific) for 30 min at RT, and incubated in primary antibodies (Supplementary Table S4) overnight at 4 °C in 5% normal serum. Sections were washed three times with PBS for 10 min each, followed by incubation with Alexa fluor-conjugated secondary antibodies (1:1000; Invitrogen) and DAPI for 1 h at RT, followed by three washes of 10 min each; they were then mounted with coverslips. Sections were visualized under AxioPlan-2 fluorescent microscope equipped with Zeiss AxioVision 4.0 software (Carl Zeiss, Oberkochen, Germany). Images were captured using Axiocam MR Camera (Carl Zeiss, Oberkochen, Germany) and analyzed with Fiji (ImageJ 1.54f, NIH, Bethesda, MD, USA) image analysis software.

### 2.6. ELISA

Following the completion of the treatments, 0.5 mL of the spent culture medium from each treatment well was collected and stored in sterile 1.5 mL low-bind microcentrifuge tubes at −20 °C until analysis. ELISA assays were performed using human PP14 (PAEP) and SPP1 ELISA kits (Thermo Fisher Scientific, Waltham, MA, USA) following the manufacturer’s instructions. For each assay, culture supernatant was thawed, and 100 µL per technical replicate was used; samples were run in triplicate. Absorbance was measured using a microplate reader (Infinite Pro200, Tecan GmbH), and protein concentrations were determined by comparing sample absorbance to a standard curve generated from known concentrations provided by the kit. The results are expressed in pg/mL. All steps were conducted at room temperature unless otherwise specified.

### 2.7. Statistical Analysis

hEOs were generated from three donor biopsies (*n* = 3), and three experimental replicates were included for each donor-derived hEOs. Three technical replicates were included from each sample for quantitative analyses (qPCR and ELISA). Data from technical replicates were averaged and aggregated for each experimental (*n* = 3) and biological (*n* = 3) replicate. Analysis was conducted with the GraphPad Prism 10 software package. Prior to hypothesis testing, data were assessed for normality using the Shapiro–Wilk test. For data that met the assumption of normality, parametric tests were used. After normalization, ∆∆Ct fold changes between the treatment and control group were calculated and analyzed by one-way ANOVA with Tukey’s multiple-comparison test and post hoc correction and two-way ANOVA with Sidak multiple comparisons. *p* < 0.05 was considered significant. ELISA detection was first plotted against known standards and analyzed with a 4-parametric curve fit model for deriving the unknown concentration and further analyzed with one-way ANOVA as mentioned above.

## 3. Results

### 3.1. Establishment of Endometrial Organoid

Endometrial organoids (hEOs) were successfully derived from endometrial biopsies obtained from three donors (Figure 1). Organoids exhibited rapid proliferation, forming glandular structures within the first two weeks of culture. The organoids were cultured in a 12-well plate-spinning bioreactor, which supports long-term 3D culture by providing continuous suspension and enhanced nutrient and oxygen diffusion. This method appeared particularly useful in extending the culture period beyond 28 days, as the BME (Geltrex) typically used for embedding organoids starts to degrade after approximately four weeks. By using the spinning bioreactor, we were able to maintain the organoids in culture for up to 44 days without the need for re-embedding in BME. This helped preserve the structural organization of the organoids, ensuring long-term viability and expansion (Figure 1D). Immunofluorescence labeling confirmed the expression of key epithelial (E-cadherin and cytokeratin) and stromal (vimentin) markers, demonstrating that the organoids retain the essential characteristics of endometrial tissue. However, the stromal cells almost disappeared by the end of the 21 days of culture (Supplementary Figure S1).

### 3.2. Human Endometrial Organoids Respond to Estrogen Treatment

**Figure 2:**
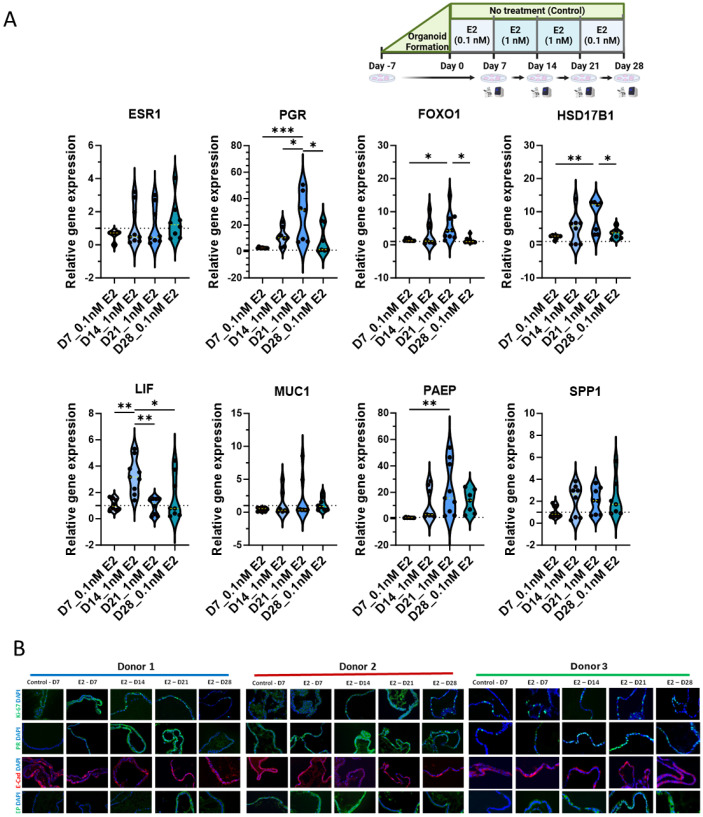
Human endometrium organoids respond to estrogen treatment: (**A**) treatment plan and violin plots of gene expression changes in hEOs during 28 days with estrogen (E2) treatment measured by quantitative polymerase chain reaction (qPCR); (**B**) representative immunofluorescent images showing, Ki67, PGR (PR), E-cadherin (E-Cad), and PAEP expression through 28-day estrogen treatment in hEOs from donor 1 (cyan), donor 2 (red), and donor 3 (green). Results illustrated in (**A**) represent mean ± SD from three independent biological replicates (*n* = 3 donors), each with three experimental replicates (*n* = 9 total) and analyzed by one-way ANOVA with Tukey’s multiple-comparison test and post hoc correction, * *p* < 0.05, ** *p* < 0.01, and *** *p* < 0.001; scale bar in (**B**): 50 µm.

Once the long-term organoid culture was established, hEOs were exposed to estradiol (E2) in a stepwise manner across four time points (days 7, 14, 21, and 28) to mimic the proliferative phase of the menstrual cycle (Figure 1C). *ESR1* (estrogen receptor alpha) expression seemed to increase from day 14 onwards, indicating a time-dependent response to E2 treatment (Figure 2A). However, the changes were not significant. *PGR* (progesterone receptor) expression showed a similar pattern, with a significant upregulation at day 21 (*p* < 0.001, *p* < 0.047) and a decrease by day 28 (*p* < 0.017), reflecting the typical estrogen-driven upregulation of PGR (Figure 2A). *FOXO1* (*p* < 0.047) and *HSD17B1* (*p* < 0.008) expression followed similar trends, increasing significantly at day 21, suggesting the induction of endometrial differentiation by E2 (Figure 2A). The expression of *PAEP*, a key marker associated with the secretory phase, also increased significantly (*p* < 0.009) at day 21, further demonstrating the responsiveness of hEOs to E2 treatment (Figure 2A). *LIF* expression peaked at day 14 (*p* < 0.003) and significantly declined by day 28 (*p* < 0.032), while no significant changes were observed in *MUC1* and *SPP1* expression despite a trend of increase at day 14 and a decline by day 28 of exposure. However, donor-specific variability in gene expression was notable. The immunohistochemical expression of Ki67, PR, PAEP, and E-cadherin (E-Cad) corroborated gene expression changes. Ki67 immunoexpression following E2 treatment until day 28 (Figure 2B) further indicated that sequential hormonal treatment can be used for endometrium organoid expansion due to the renewal and proliferation of glandular epithelial cells that also express E-cadherin throughout. These results highlight the ability of hEOs to recapitulate the proliferative phase in response to estradiol.

### 3.3. Sequential Estrogen and Progesterone Treatment Mimics the Secretory Phase

**Figure 3:**
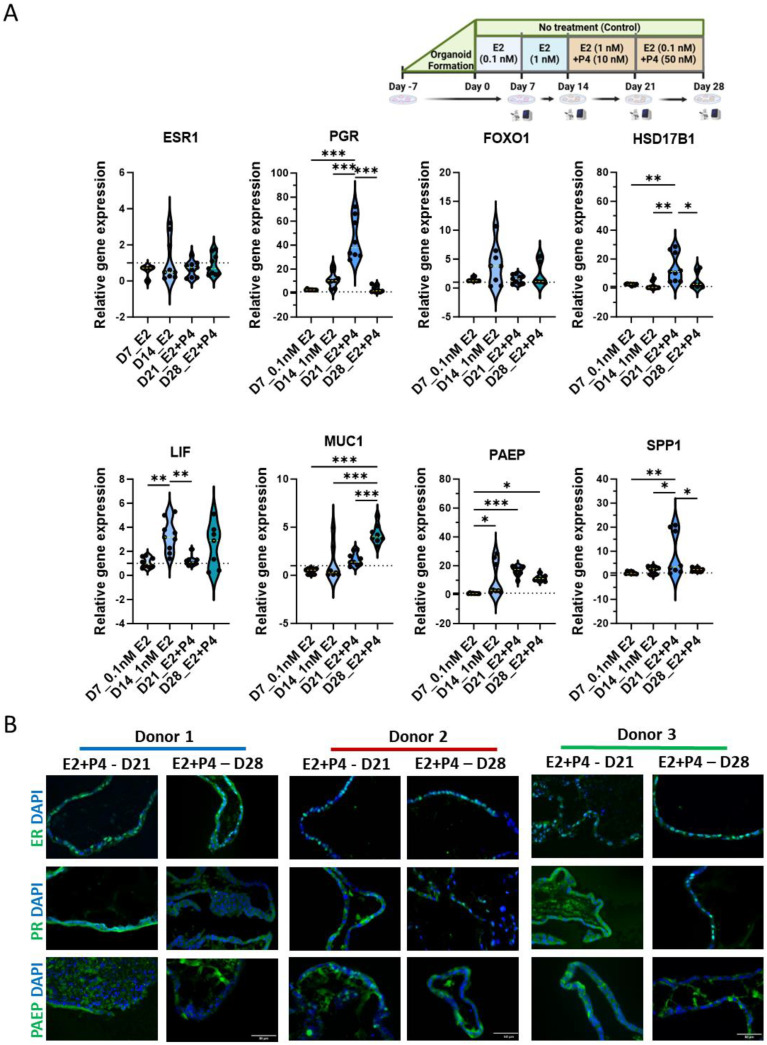
Sequential estrogen and progesterone treatment mimics the secretory phase in human endometrial organoids: (**A**) treatment plan and violin plots of gene expression changes in hEOs during 28 days with sequential progesterone (P4) treatment following estrogen (E2) day 14 onwards, measured by quantitative polymerase chain reaction (qPCR); (**B**) representative immunofluorescent images showing ESR1 (ER), PGR (PR), and PAEP expression through 28-day treatment in hEOs from donor 1 (cyan), donor 2 (red), and donor 3 (green). Results illustrated in (**A**) represent mean ± SD from three independent biological replicates (*n* = 3 donors), each with three experimental replicates (*n* = 9 total), and analyzed by one-way ANOVA with Tukey’s multiple-comparison test and post hoc correction, * *p* < 0.05, ** *p* < 0.01, and *** *p* < 0.001; scale bar in (**B**): 50 µm.

To simulate the transition from the proliferative to the secretory phase, hEOs were treated with both estradiol and progesterone (P4) from day 14 onwards (Figure 1C). The combined hormone treatment led to significantly high expression of *PGR* at day 21 (*p* < 0.001), decreasing by day 28 to untreated levels (*p* < 0.001) (Figure 3A). *HSD17B1* expression followed similar trends, increasing significantly at day 21 (*p* < 0.027), which suggests the induction of endometrial differentiation by E2 (Figure 3A). *FOXO1*, another marker of endometrial differentiation, also exhibited a trend of increased expression at day 14, followed by a reduction by day 21; however, the changes were not significant (Figure 3A). *LIF* expression peaked at day 14 (*p* < 0.003), with a decline at day 21 (*p* < 0.005), while *MUC1* was significantly increased only by day 28 (*p* < 0.001). *PAEP* and *SPP1*, important markers of the mid-secretory phase, were markedly elevated at day 21 (*p* < 0.001 and *p* < 0.008) in response to progesterone; however, their expression declined by day 28, signaling the end of the secretory phase (*p* < 0.049) (Figure 3A). Immunolabeling further confirmed that ER, PR, and PAEP protein levels corresponded with gene expression changes, supporting the gene-level findings (Figure 3B). These results indicate that sequential hormone treatment successfully mimics the dynamic hormone-driven changes in the menstrual cycle in hEOs.

### 3.4. cAMP Enhances Progesterone-Induced Differentiation

**Figure 4:**
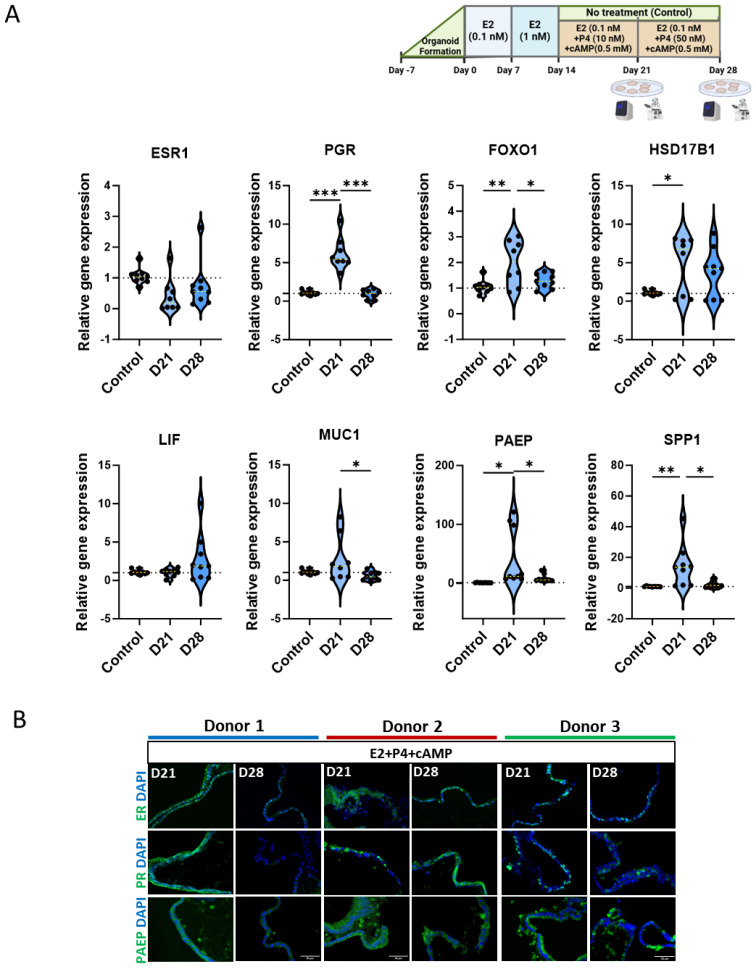
cAMP enhances progesterone-induced differentiation of human endometrial organoids: (**A**) treatment plan and violin plots of gene expression changes in hEOs during 28 days with sequential progesterone (P4) and cAMP treatment following estrogen (E2) treatment from day 14 onwards in hEOs measured by quantitative polymerase chain reaction (qPCR); (**B**) representative immunofluorescent images showing ESR1 (ER), PGR (PR), and PAEP expression at day 21 and 28 through 28-day treatment in hEOs from donor 1 (cyan), donor 2 (red), and donor 3 (green). Results illustrated in (**A**) represent mean ± SD from three independent biological replicates (*n* = 3 donors), each with three experimental replicates (*n* = 9 total), and analyzed by one-way ANOVA with Tukey’s multiple-comparison test and post hoc correction, * *p* < 0.05, ** *p* < 0.01, and *** *p* < 0.001; scale bar in (**B**): 50 µm.

In addition to sequential estrogen (E2) and progesterone (P4) treatments, we investigated the effects of cAMP, a key decidualization agent, on the differentiation of human endometrial organoids (hEOs). Starting from day 14, hEOs were treated with 0.5 mM cAMP alongside E2 and P4 (EPC treatment) until day 28, and gene expression was analyzed at days 21 and 28 (Figure 4A). The expression of hormone receptors and differentiation markers was significantly altered by cAMP treatment.

*ESR1* (estrogen receptor alpha) expression remained consistently below basal levels. In contrast, *PGR* (progesterone receptor) expression significantly increased at day 21 (*p* < 0.001) and declined by day 28 (*p* < 0.001), returning to control levels (Figure 4A). A similar trend in gene expression was observed for *FOXO1*, *HSD17B1*, and *MUC1* with a significant increase at day 21 (*p* < 0.004, *p* < 0.027, and *p* < 0.039, respectively), while no significant changes were observed for *LIF* expression (Figure 4A).

Markers of endometrial differentiation, namely *PAEP* (progestagen-associated endometrial protein) and *SPP1* (secreted phosphoprotein 1), showed a substantial increase by day 21 (*p* < 0.013 and *p* < 0.007) in response to EPC treatment. Both *PAEP* and *SPP1* expression decreased by day 28 (*p* < 0.044 and *p* < 0.015), returning to levels comparable to untreated control hEOs (Figure 4A). These data suggest that cAMP strongly promotes mid-secretory differentiation but loses its effect by day 28. Immunohistochemistry for ER, PR, and PAEP at days 21 and 28 confirmed the gene expression results, demonstrating that the protein levels corresponded well with transcript changes (Figure 4B). *ESR1* expression was generally lower in EPC-treated hEOs compared to controls, while *PGR* and *PAEP* showed peak expression at day 21 and diminished by day 28.

**Figure 5:**
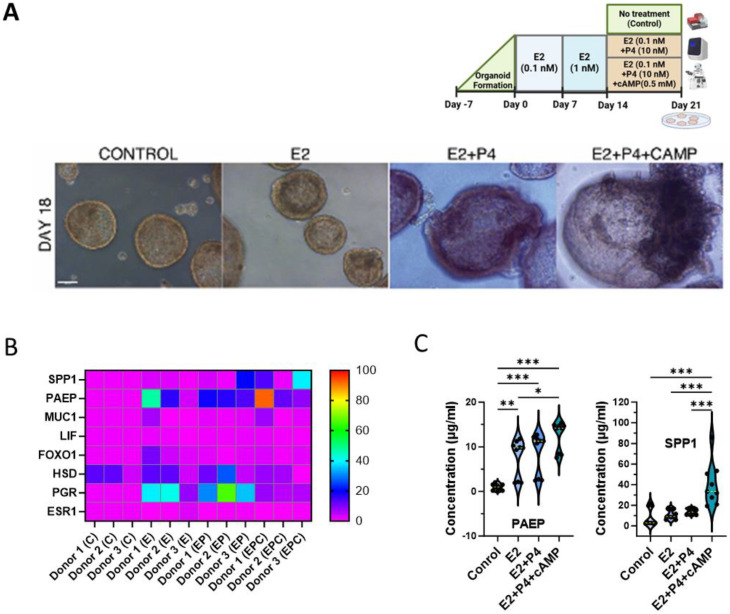
Human endometrium organoids treated with sequential estrogen and progesterone with cAMP induce the secretory phase: (**A**) treatment plan and bright field images of endometrial organoids cultured at 18 days in vitro with different hormone treatments in hEOs; (**B**) heatmap of gene expression changes at day 21 in hEOs measured by quantitative polymerase chain reaction (qPCR); (**C**) violin plot of ELISA for secreted PAEP (left) and SPP1 (right) from culture supernatant with different hormone treatments at day 21 in hEOs. Results illustrated in (**B**) represent mean ± SD (*n* = 3) and were analyzed by two-way ANOVA with Sidak multiple comparisons; the results in C represent mean ± SD from three independent biological replicates (*n* = 3 donors), each with three experimental replicates (*n* = 9 total), and analyzed by one-way ANOVA with Tukey’s multiple-comparison test and post hoc correction, * *p* < 0.05, ** *p* < 0.01, and *** *p* < 0.001; scale bar in (A): 50 µm.

The morphological changes observed in hEOs further supported these findings. By day 18, control and E2-only-treated hEOs displayed compact structures with smooth boundaries, whereas E2 + P4 (EP) and E2 + P4 + cAMP (EPC)-treated organoids appeared larger, with fluid-filled cavities and budding structures (Figure 5A). These morphological changes are indicative of advanced differentiation, resembling the mid- to late-secretory phase in vivo.

Since significantly high differences in gene expression were observed mainly at day 21 following EPC treatment (Figure 4A), we compared gene expression between E2 only (E), E2 with P4 (EP), and EP with cAMP (EPC)-treated hEOs at day 21. *ESR1* expression was relatively at the lowest level in all treatment groups at D21 (Figure 5B and Figure S2). Though *PGR* expression was already upregulated in the E2-only group, the expression further increased following progesterone treatment (EP) with a decline in EPC-treated hEOs. *FOXO1*, *LIF*, and *MUC1* expression did not change significantly following EP and EPC treatment compared to E2-only-treated hEOs (Figure 5B and Figure S2). *HSD17B1* expression was generally higher in E2-only and EP treatments compared to EPC-treated hEOs. Surprisingly *PAEP* expression was remarkably higher in the E2-only-treated group compared to untreated hEOs and remained highly expressed with EP and EPC treatment, while SPP1 expression was significantly upregulated in EPC-treated hEOs (Figure 5B and Figure S2). Furthermore, donor-specific differences were observed in the gene expression of hEOs, indicating donor variability (Supplementary Figure S2).

### 3.5. Secretory Protein Secretion Validates Functional Differentiation

To confirm the functional differentiation of hEOs into a secretory state, ELISA was performed to quantify the secretion of uterine milk proteins PAEP and SPP1 from culture supernatants. Both proteins were significantly elevated at day 21 in the E2 + P4 (*p* < 0.001) and E2 + P4 + cAMP groups (*p* < 0.001), with the highest secretion observed in the E2 + P4 + cAMP group (Figure 5C). This indicates that the organoids effectively recapitulated the secretory phase of the menstrual cycle, with cAMP further enhancing their secretory function. The elevated secretion of PAEP and SPP1 at day 21 closely mimics the in vivo environment of the mid- to late-secretory phase, confirming the functional differentiation of organoids in response to hormone treatments.

## 4. Discussion

We attempted to derive human endometrial organoids (hEOs) from endometrial biopsies of proliferative phase endometrium. We satisfactorily showed that endometrial organoids can be derived from donor biopsies and cultured long term, and they efficiently mimic the complete 28 days of the menstrual cycle, when exposed to doses of estrogen and progesterone resembling in vivo conditions. These hEOs closely mimic gene expression patterns of proliferative and secretory phases of human endometrium, including *PGR*, *HSD17B1*, *MUC1*, *PAEP*, and *SPP1*, upon hormone stimulation. We dissected the effect of individual hormonal regimens in hEOs until the end of the treatment cycle. We further detected high levels of secreted factors of uterine milk in culture supernatants such as PAEP (glycodelin) and SPP1 (osteopontin) at day 21 that recapitulate mid- to late-secretory phase. These findings align with advancements in organoid technology [11,12,13,24,25,26,27,28,29], confirming the robustness of hEOs as reliable models for studying endometrial biology over extended periods. We analyzed the responsiveness of these organoids to the sequential treatment of estrogen starting with low-to-high-to-low doses to mimic in vivo estrogen levels. ESR1 expression in our hEOs remained at basal levels following E2 treatment, while Ki67-positive cells were evident at all treatment days. Moreover, around days 14 and 21, these hEOs exhibited increased expression of PGR, indicating a response similar to the in vivo estrogen-induced sensitization of the endometrium before the secretory phase. Additionally, other markers such as *LIF*, *FOXO1*, *HSD17B1*, *PAEP*, and *SPP1* were also elevated. Furthermore, the addition of progesterone treatment from day 14 onwards led to an enhanced upregulation of PGR and other differentiation markers such as PAEP and SPP1 until day 21, followed by a decline at the end of treatment day 28. However, this pattern is comparable to the hormone responses observed in previously reported studies but also differs in temporal gene expression patterns, underscoring the importance of physiological timing and doses of hormone treatments to mimic in vivo endometrial responses [13,24,30,31]. In a seminal study on biopsy-derived endometrial organoids and their responsiveness to hormones [13], endometrial organoids were primed with 10 nM E2 for 48 h followed by 1 μM P4 for 4 days, and gene expression changes were reported; however, the study did not focus on mimicking complete menstrual cycle. Luddi et al. (2020) also established endometrial organoids to investigate the endometrial–embryo interaction, particularly during the implantation window, focusing on secretory markers such as glycodelin to assess endometrial receptivity [32]. While this work demonstrated the applicability of organoids for implantation studies, it did not explore long-term hormonal dynamics across the menstrual cycle. Boretto et al. [30] developed organoids from mouse and human endometrium and treated either with E2 (1 nM) only for 7 days or followed by P4 (50 ng/mL) for another 7 days. Their study was largely focused on the gene expression of WNT and LGR-associated genes and did not provide a complete overview of the temporal expression of E2- and P4-responsive genes, nor a culture for over 28 days. Another study by Haider et al. [33] investigated ciliogenesis in human endometrial organoids in response to 10 nM β-estradiol and analyzed the expression of ciliary structural genes. Boretto et al. [34] further showed the feasibility of generating expandable organoids from a broad spectrum of endometrial pathologies recapitulating disease-associated traits and cancer-linked mutations. However, their study did not include physiological hormonal influences per se. Luongo et al. (2023) explored organoids derived from menstrual blood and assessed their potential for implantation studies. While they observed responsiveness to short-term hormonal treatments, their study was limited to the secretory phase and did not explore hormone responses beyond the implantation window, nor did it model the full 28-day menstrual cycle [35]. Wiwatpanit et al. [36] showed the effect of increased androgen levels (in PCOS) on the endometrium utilizing novel scaffold-free multicellular endometrial organoids. These hEOs showed increased cell proliferation and dysregulated gene expression upon 14 days of E2 and testosterone treatment. It should be noted that timed mid-luteal progesterone levels are nearly 10 ng/mL; however, earlier studies have used P4 doses well beyond this range. Moreover, none of the studies so far have reported the responsiveness of hEOs over 28 days of hormone treatment and how this could affect gene expression. Our study, on the other hand, investigated a 28-day physiological hormone treatment and analyzed temporal changes in gene expression levels at each stage for the key transcription factors and receptors. We also tested if cAMP, often used as a differentiation factor for decidualization, could further enhance the secretory phenotype of these hEOs. The addition of cAMP alongside estrogen and progesterone from day 14 onwards induced *PAEP* (glycodelin) and *SPP1* (osteopontin) gene expression and secretion in culture supernatant by day 21, aligning with other studies [13,24,31]. However, we noticed interindividual differences in the gene expression of hEOs from different donor biopsies, but overall response to the hormonal treatment showed the ability of hEOs to sequentially mimic the endometrial proliferative and secretory phase. These differences between samples could be attributable to ongoing systemic or pathological conditions or due to epigenetic differences between donors [11,25]. Nevertheless, variability attributable to culture conditions should not be disregarded. We employed a serum-free expansion medium widely utilized in numerous studies [12,13,24]. Surprisingly, we noticed that N2 and B27 supplements, which are key components of reported hEO media, contain considerably high levels of progesterone, amounting to 70 nM [37,38,39]. We further suspect that such high levels of progesterone in culture media could explain the precocious expression of *PGR*, *PAEP*, and *SPP1* observed in hEOs already starting at day 14 and saturated by day 21, indicating synchronization to the early secretory phase. Although others have reported the effect of progesterone treatment on hEO differentiation and decidualization using a similar widely used defined serum-free media, these treatments were of shorter durations, ranging from 2 days up to a week [13,24], with a very high dose of progesterone (up to 1 ug/mL or 1µM). This may have shown a positive effect in short-term culture, but it is well beyond the physiological range of hormone concentration in vivo in humans [40]. The diversity of observed results across laboratories highlights the need for carefully considering culture conditions and donor biological variability when modeling hormone-sensitive tissue-derived organoids. This aspect of variability and hormonal influence is a critical theme in endometrial organoid research, especially in the study of 28-day hormone treatment models. Our findings, in the context of other studies, suggest that hEOs offer a promising avenue for exploring the dynamics of endometrial receptivity, particularly in endometrial pathologies like infertility or endometriosis. Earlier reports on the actions of estrogen and progesterone [41] provide further insight into the potential pathological conditions where hormonal responsiveness may be altered, suggesting new directions for future research in this field. Although the hEOs in this study were of epithelial origin, we observed some residual stromal cells during early culture (e.g., day 7), which disappeared by day 21. This aligns with previous studies suggesting that stromal cells require different conditions for survival, such as nutrient needs or adherence to the culture dish rather than remaining within the BME/Matrigel domes. Recent advances in endometrial modeling have incorporated stromal cells into endometrial assembloids, combining both epithelial and stromal populations to better replicate in vivo conditions [42,43]. However, the loss of stromal cells in extended cultures remains a significant limitation, as the stromal compartment plays a critical role in processes like decidualization, as well as in conditions such as recurrent implantation failure (RIF) and endometriosis. Our study focused on mimicking the proliferative and secretory phases of the 28-day menstrual cycle to assess molecular responses in the epithelial compartment. The withdrawal phase, which involves the shedding of the endometrial lining, was not modeled, as it would require a more complex system incorporating stromal cells and spiral arteries, which are critical for the menstruation and repair processes. Future refinements of the organoid model could integrate these components to more accurately simulate the entire menstrual cycle, including the withdrawal phase. We acknowledge the limitations of the small sample size in our study (*n* = 3), potentially affecting type 2 errors and the generalizability of findings across diverse patient populations. However, this is a preliminary investigation to explore feasibility, refine protocols, or generate hypotheses, with the understanding that these results will need confirmation in larger, more definitive studies. Moreover, our study depicts a highly controlled culture condition where the technical and experimental variability is extremely low, and the expected effect size is very large; a smaller sample size might still yield meaningful results for larger studies in the future. We carefully selected putative markers of proliferative and secretory phase endometrium to follow temporal changes in expression since these markers have been widely discussed and reported earlier and serve as reliable readouts for characterization. We suggest that future investigations in disease-associated endometrial organoids should include an unbiased RNA sequencing or proteomic approach to find novel biomarkers or therapeutic targets. Although our study primarily focuses on the hormonal aspect, future investigations warrant the possible inclusion of other factors influencing endometrial health and disease. We have pointed out the careful consideration of culture media components to avoid excessively high levels of progesterone that could otherwise prime the endometrial response to the secretory phase. This highlights the complexities of hormonal regulation and individual variability, pointing toward the need for personalized approaches in organoid-based research and potential applications in understanding and treating reproductive pathologies. Our findings further propose that hEOs offer a promising avenue for exploring the dynamics of endometrial receptivity, particularly in the context of endometrial pathologies like infertility or endometriosis, and provide further opportunities to gain insight into the potential pathological conditions where hormonal responsiveness may be altered, suggesting new directions for biomarker discovery and therapeutic interventions in future research in this field.

## 5. Conclusions

Our study represents a significant advance in the field of reproductive biology, providing comprehensive insights into the behavior of human endometrial organoids (hEOs) under a sequential hormonal treatment regimen that mimics a 28-day menstrual cycle. hEOs are sustainable in long-term cultures and respond to fluctuating hormone levels, reflecting characteristics of both the proliferative and secretory phases of the endometrium. The observed variations in gene expression among organoids derived from different donors highlight the potential for hEOs in personalized medicine, particularly for addressing reproductive pathologies. Importantly, our findings also draw attention to the need for careful consideration of culture conditions, especially the influence of media components often consisting of high levels of progesterone. This research paves the way for future optimization of culture conditions and studies to utilize hEOs as a model system for endometrial diseases, therapeutic development, and regenerative medicine, advancing our understanding of endometrial biology and its role in female reproductive health, leading to more effective and individualized patient care in obstetrics and gynecology.

## Data Availability

Data regarding any of the subjects in the study have not been previously published unless specified. Data will be made available upon query or request.

## Supplementary Materials

The following supporting information can be downloaded at: https://www.mdpi.com/article/10.3390/cells13211811/s1, Figure S1: Human endometrium organoids continuously express key epithelial marker. A, Immunofluorescent images showing Cytokeratin (epithelial cell marker), and Vimentin (stromal cell marker) expression through 28-day estrogen treatment in donor 3 derived organoids. Control (day 7), E2 (day 14), E2 + P4 (day 21), E2 + P4 + cAMP (day 28). Nuclei counterstained in blue with DAPI Scale bar: 50um; Figure S2: Human endometrium organoid treated with sequential estrogen and progesterone with cAMP represent donor variability. Gene expression changes at day 21 in hEOs measured by quantitative polymerase chain reaction (qPCR) showing 3 donor samples individually. Results illustrated represent mean ± SD from three experimental replicates (*n* = 3 total) and analyzed by two-way ANOVA with Sidak multiple comparisons, * *p* < 0.05, ** *p* < 0.01, and *** *p* < 0.001; Figure S3: Immunostaining negative control for human endometrium organoid at culture day 28. 7 µm thick cryosections of hEOs were immunostained with fluorophore labelled secondary antibodies; goat anti rabbit Alexa fluor 488 (green, upper panel) and goat anti mouse Alexa fluor 568 (red, lower panel) while omitting the antigen-specific primary antibodies to assess the non-specific staining. Scale bar: 50um; Table S1: Composition of tissue collection/wash medium and Enzymatic tissue digestion mixture for glandular epithelia isolation from scratch biopsies; Table S2: Composition of human endometrial organoid expansion medium (hEOM) and details of hormonal treatments used in the study; Table S3: Oligonucleotide primer sequences used for RT-qPCR analysis; Table S4: Details of antibodies and dilutions used in the study.

## References

[1] Gargett C.E., Chan R.W., Schwab K.E. (2008). Hormone and growth factor signaling in endometrial renewal: Role of stem/progenitor cells. Mol. Cell. Endocrinol..

[2] Töhönen V., Antonson P., Boggavarapu N.R., Ali H., Motaholi L.A., Gustafsson J.Å., Varshney M., Rodriguez-Wallberg K.A., Katayama S., Nalvarte I. (2024). Transcriptomic profiling of the oocyte-cumulus-granulosa cell complex from estrogen receptor β knockout mice. F S Sci..

[3] Smith E.S., Moon A.S., O’Hanlon R., Leitao M.M., Sonoda Y., Abu-Rustum N.R., Mueller J.J. (2020). Radical Trachelectomy for the Treatment of Early-Stage Cervical Cancer: A Systematic Review. Obs. Gynecol..

[4] Rodriguez-Wallberg K.A., Waterstone M., Anastácio A. (2019). Ice age: Cryopreservation in assisted reproduction—An update. Reprod. Biol..

[5] Cao Y., Sun H., Zhu H., Zhu X., Tang X., Yan G., Wang J., Bai D., Wang J., Wang L. (2018). Allogeneic cell therapy using umbilical cord MSCs on collagen scaffolds for patients with recurrent uterine adhesion: A phase I clinical trial. Stem Cell Res. Ther..

[6] Chang Z., Zhu H., Zhou X., Zhang Y., Jiang B., Li S., Chen L., Pan X., Feng X.L. (2021). Mesenchymal Stem Cells in Preclinical Infertility Cytotherapy: A Retrospective Review. Stem Cells Int..

[7] Gargett C.E., Ye L. (2012). Endometrial reconstruction from stem cells. Fertil. Steril..

[8] Lancaster M.A., Knoblich J.A. (2014). Organogenesis in a dish: Modeling development and disease using organoid technologies. Science.

[9] Xiao S., Coppeta J.R., Rogers H.B., Isenberg B.C., Zhu J., Olalekan S.A., McKinnon K.E., Dokic D., Rashedi A.S., Haisenleder D.J. (2017). A microfluidic culture model of the human reproductive tract and 28-day menstrual cycle. Nat. Commun..

[10] Turco M.Y., Moffett A. (2019). Development of the human placenta. Development.

[11] Brucker S.Y., Hentrich T., Schulze-Hentrich J.M., Pietzsch M., Wajngarten N., Singh A.R., Rall K., Koch A. (2022). Endometrial organoids derived from Mayer-Rokitansky-Küster-Hauser syndrome patients provide insights into disease-causing pathways. Dis. Model. Mech..

[12] Katcher A., Yueh B., Ozler K., Nizam A., Kredentser A., Chung C., Frimer M., Goldberg G.L., Beyaz S. (2023). Establishing patient-derived organoids from human endometrial cancer and normal endometrium. Front. Endocrinol..

[13] Turco M.Y., Gardner L., Hughes J., Cindrova-Davies T., Gomez M.J., Farrell L., Hollinshead M., Marsh S.G.E., Brosens J.J., Critchley H.O. (2017). Long-term, hormone-responsive organoid cultures of human endometrium in a chemically defined medium. Nat. Cell Biol..

[14] Bulun S.E., Adashi E., Kronenberg H.M., Melmed S., Polonsky K.S., Larsen P.R. (2007). The Physiology and Pathology of the Female Reproductive Axis. Williams Textbook of Endocrinology.

[15] Scholzen T., Gerdes J. (2000). The Ki-67 protein: From the known and the unknown. J. Cell Physiol..

[16] Patel B., Elguero S., Thakore S., Dahoud W., Bedaiwy M., Mesiano S. (2015). Role of nuclear progesterone receptor isoforms in uterine pathophysiology. Hum. Reprod. Update.

[17] Mustonen M., Isomaa V., Vaskivuo T., Tapanainen J., Poutanen M., Stenbäck F., Vihko R., Vihko P. (1998). Human 17β-Hydroxysteroid Dehydrogenase Type 2 Messenger Ribonucleic Acid Expression and Localization in Term Placenta and in Endometrium during the Menstrual Cycle1. J. Clin. Endocrinol. Metab..

[18] Evans J., Salamonsen L.A., Winship A., Menkhorst E., Nie G., Gargett C.E., Dimitriadis E. (2016). Fertile ground: Human endometrial programming and lessons in health and disease. Nat. Rev. Endocrinol..

[19] Kim J.J., Buzzio O.L., Li S., Lu Z. (2005). Role of FOXO1A in the regulation of insulin-like growth factor-binding protein-1 in human endometrial cells: Interaction with progesterone receptor. Biol. Reprod..

[20] Seppälä M., Taylor R.N., Koistinen H., Koistinen R., Milgrom E. (2002). Glycodelin: A major lipocalin protein of the reproductive axis with diverse actions in cell recognition and differentiation. Endocr. Rev..

[21] Dey S.K., Lim H., Das S.K., Reese J., Paria B.C., Daikoku T., Wang H. (2004). Molecular cues to implantation. Endocr. Rev..

[22] Gellersen B., Brosens J.J. (2014). Cyclic decidualization of the human endometrium in reproductive health and failure. Endocr. Rev..

[23] Qian X., Jacob F., Song M.M., Nguyen H.N., Song H., Ming G.L. (2018). Generation of human brain region-specific organoids using a miniaturized spinning bioreactor. Nat. Protoc..

[24] Fitzgerald H.C., Dhakal P., Behura S.K., Schust D.J., Spencer T.E. (2019). Self-renewing endometrial epithelial organoids of the human uterus. Proc. Natl. Acad. Sci. USA.

[25] Smirnov A., Melino G., Candi E. (2023). Gene expression in organoids: An expanding horizon. Biol. Direct.

[26] Maenhoudt N., De Moor A., Vankelecom H. (2022). Modeling Endometrium Biology and Disease. J. Pers. Med..

[27] Zhou W., Barton S., Cui J., Santos L.L., Yang G., Stern C., Kieu V., Teh W.T., Ang C., Lucky T. (2022). Infertile human endometrial organoid apical protein secretions are dysregulated and impair trophoblast progenitor cell adhesion. Front. Endocrinol..

[28] Jiang Y., Varshney M., Palomares A.R., Inzunza J., Rodriguez-Wallberg K.A. (2023). Gene expression of human endometrial organoids hormonally treated in vitro for 28 days. Hum. Reprod..

[29] Jiang Y., Varshney M., Palomares A.R., Inzunza J., Acharya G., Nalvarte I., Rodriguez-Wallberg K.A. (2023). Functional Assessment of Human Endometrial Organoid Responsiveness to a Sequentially Controlled Hormonal Treatment Mimicking Menstrual Cycle. Fertil. Steril..

[30] Boretto M., Cox B., Noben M., Hendriks N., Fassbender A., Roose H., Amant F., Timmerman D., Tomassetti C., Vanhie A. (2017). Development of organoids from mouse and human endometrium showing endometrial epithelium physiology and long-term expandability. Development.

[31] Olalekan S.A., Burdette J.E., Getsios S., Woodruff T.K., Kim J.J. (2017). Development of a novel human recellularized endometrium that responds to a 28-day hormone treatment. Biol. Reprod..

[32] Luddi A., Pavone V., Semplici B., Governini L., Criscuoli M., Paccagnini E., Gentile M., Morgante G., Leo V., Belmonte G. (2020). Organoids of Human Endometrium: A Powerful In Vitro Model for the Endometrium-Embryo Cross-Talk at the Implantation Site. Cells..

[33] Haider S., Gamperl M., Burkard T.R., Kunihs V., Kaindl U., Junttila S., Fiala C., Schmidt K., Mendjan S., Knöfler M. (2019). Estrogen Signaling Drives Ciliogenesis in Human Endometrial Organoids. Endocrinology.

[34] Boretto M., Maenhoudt N., Luo X., Hennes A., Boeckx B., Bui B., Heremans R., Perneel L., Kobayashi H., Van Zundert I. (2019). Patient-derived organoids from endometrial disease capture clinical heterogeneity and are amenable to drug screening. Nat. Cell Biol..

[35] Luongo F.P., Ortega Baño I., Morgante G., Luddi A., Piomboni P. (2023). P-803 3D human endometrium from noninvasively retrieved primary endometrial cells is a reliable model to mimic the implantation window. Hum. Reprod..

[36] Wiwatpanit T., Murphy A.R., Lu Z., Urbanek M., Burdette J.E., Woodruff T.K., Kim J.J. (2020). Scaffold-Free Endometrial Organoids Respond to Excess Androgens Associated With Polycystic Ovarian Syndrome. J. Clin. Endocrinol. Metab..

[37] Bottenstein J.E., Sato G.H. (1979). Growth of a rat neuroblastoma cell line in serum-free supplemented medium. Proc. Natl. Acad. Sci. USA.

[38] Bottenstein J.E. (1985). Cell Culture in the Neurosciences.

[39] Brewer G.J., Torricelli J.R., Evege E.K., Price P.J. (1993). Optimized survival of hippocampal neurons in B27-supplemented Neurobasal, a new serum-free medium combination. J. Neurosci. Res..

[40] Carmina E., Stanczyk F.Z., Lobo R.A., Jerome F.S., Robert L.B. (2019). Evaluation of Hormonal Status. Yen and Jaffe’s Reproductive Endocrinology (Eighth Edition).

[41] Young S.L. (2013). Oestrogen and progesterone action on endometrium: A translational approach to understanding endometrial receptivity. Reprod. Biomed. Online.

[42] Beaussart C., Rossi M., Stratopoulou C.A., Zipponi M., Cacciottola L., Donnez J., Dolmans M.M. (2024). Refining endometrial assembloids: A novel approach to 3D culture of the endometrium. F S Sci..

[43] Stratopoulou C.A., Rossi M., Beaussart C., Zipponi M., Camboni A., Donnez J., Dolmans M.M. (2024). Generation of epithelial-stromal assembloids as an advanced in vitro model of impaired adenomyosis-related endometrial receptivity. Fertil. Steril..

