# Supplementary material for: Proof-of-Concept for Long-Term Human Endometrial Epithelial Organoids in Modeling Menstrual Cycle Responses
Source: Cells. 2024 Nov 2;13(21):1811. doi: 10.3390/cells13211811 (PMC11545391; doi:10.3390/cells13211811)
Supplement: Supplementary file 1 [file cells-13-01811-s001.zip › cells-3274363-supplementary.pdf]

cells
MDPI

Supplementary Figure 1

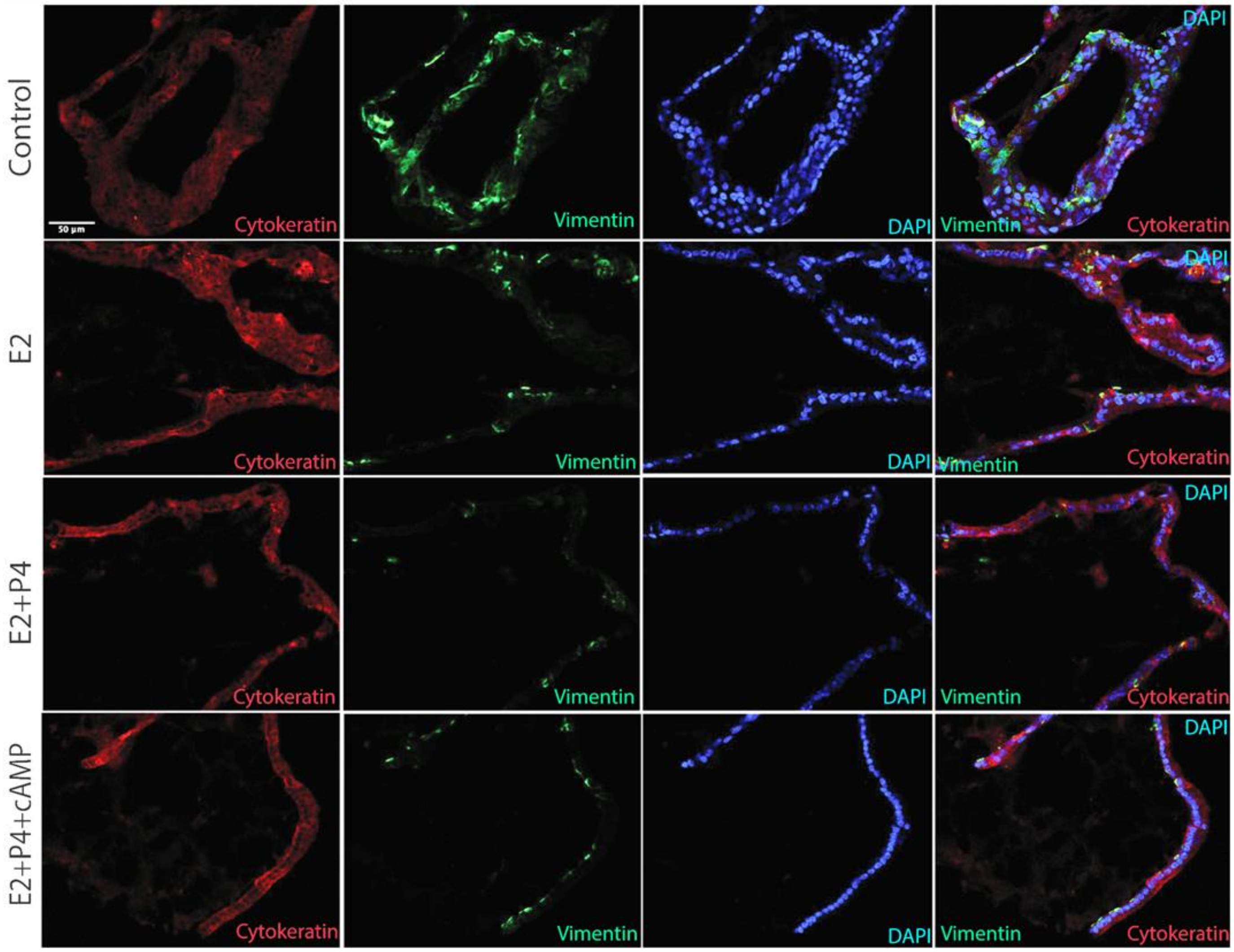

Supplementary Figure S1. Human endometrium organoids continuously express key epithelial marker. A, Immunofluorescent images showing Cytokeratin (epithelial cell marker), and Vimentin (stromal cell marker) expression through 28-day estrogen treatment in donor 3 derived organoids. Control (day 7), E2 (day 14), E2+P4 (day 21), E2+P4+cAMP (day 28). Nuclei counterstained in blue with DAPI Scale bar: 50um.

Supplementary Figure 2

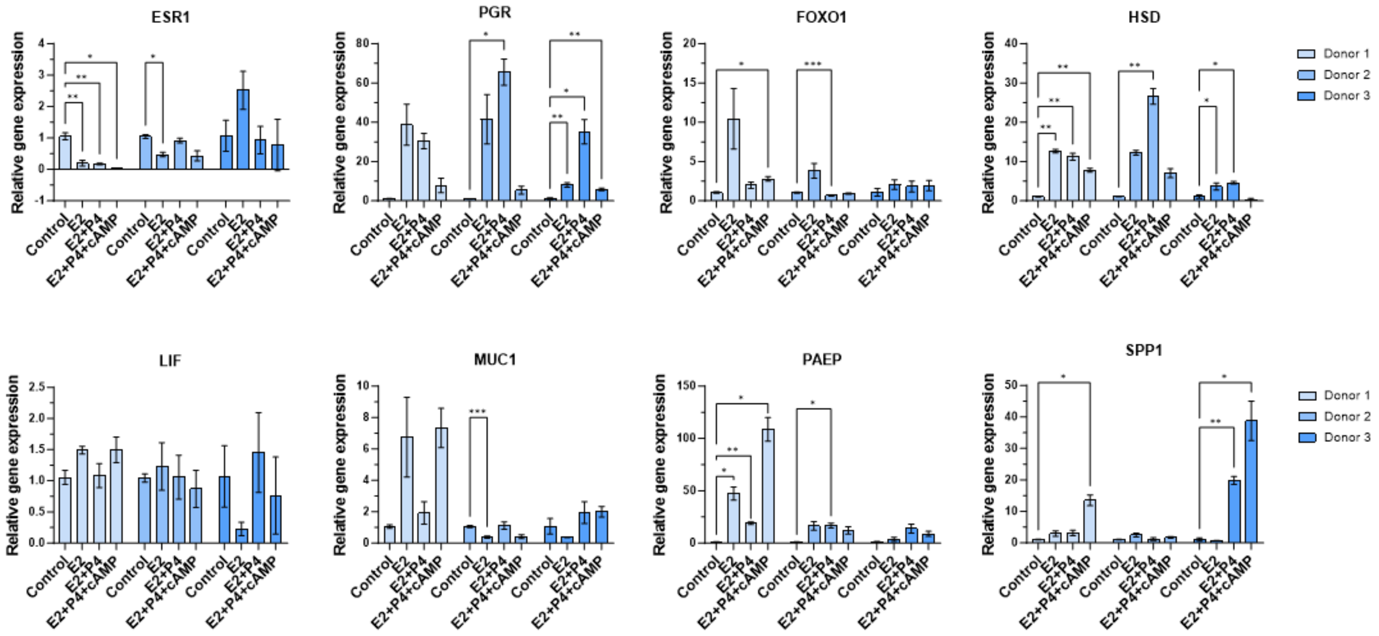

Supplementary Figure S2. Human endometrium organoid treated with sequential estrogen and progesterone with cAMP represent donor variability. Gene expression changes at day 21 in hEOs measured by quantitative polymerase chain reaction (qPCR) showing 3 donor samples individually. Results illustrated represent mean ± SD from three experimental replicates (n=3 total) and analyzed by two-way ANOVA with Sidak multiple comparisons, *P < 0.05, **P < 0.01, and ***P < 0.001.

Supplementary Figure 3

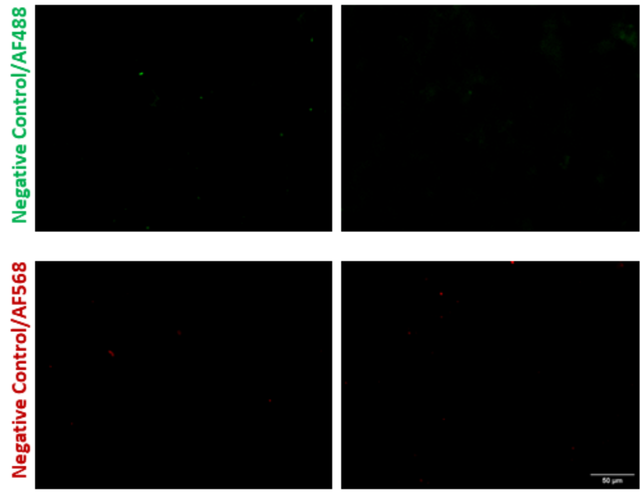

Supplementary Figure S3. Immunostaining negative control for human endometrium organoid at culture day 28. 7 µm thick cryosections of hEOs were immunostained with fluorophore labelled secondary antibodies; goat anti rabbit Alexa fluor 488 (green, upper panel) and goat anti mouse Alexa fluor 568 (red, lower panel) while omitting the antigen-specific primary antibodies to assess the non-specific staining. Scale bar: 50um

**Supplementary Table S1: Composition of tissue collection/wash medium and Enzymatic tissue digestion mixture for glandular epithelia isolation from scratch biopsies**			
**Tissue collection / Wash media**			
Product	Company	Product Number	Final Concentration
FBS	Thermo Fisher	26140-079	10%
HEPES (1M)	Thermo Fisher	15630080	10 mM
Primocin	Invivogen	ant-pm-1	1X
RPMI 1640	Gibco/Life Technologies	21875-034	to reconstitute
**Enzymatic tissue digestion mix**			
Product	Company	Product Number	Final Concentration
Collagenase	Sigma Aldrich	C-9263	2mg/ml
Dispase	Sigma Aldrich	D4693	25U/ml
DNAse I	Sigma Aldrich	4536282001	100U/uµ
FBS	Thermo Fisher	26140-079(500ml)	0.1
RPMI-1640	Gibco/Life Technologies	21875-034	to reconstitute

Supplementary Table S1. Composition of tissue collection/wash medium and Enzymatic tissue digestion mixture for glandular epithelia isolation from scratch biopsies.

**Supplementary Table S2: Composition of human endometrial organoid expansion medium (hEOM) and details of hormonal treatments used in the study**			
**Human endometrial organoid expansion medium**			
Product	Company	Product Number	Final Concentration
Advanced DMEM/F12	Life Technologies	12634010	1X
N2 supplement	Life Technologies	17502048	1X
B27 supplement minus vitamin A	Life Technologies	12587010	1X
Primocin	Invivogen	ant-pm-1	100 μg/ml
N-Acetyl-L-cysteine	Sigma Aldrich	A9165-5G	1.25 mM
Glutamax	Life Technologies	35050061	2 mM
Recombinant human EGF	Peprotech	AF-100-15	50 ng/ml
Recombinant human Noggin	Peprotech	120-10c	100 ng/ml
Recombinant human Rspondin-1	Peprotech	120-38	500 ng/ml
Recombinant human FGF-10	Peprotech	100-26	100 ng/ml
Recombinant human HGF	Peprotech	100-39	50 ng/ml
ALK-4, -5, -7 inhibitor, A83-01	Sigma Aldrich	SML0788-5MG	500 nM
Nicotinamide	Sigma Aldrich	N0636	10 nM
**Hormones used for differentiation of human endometrial organoids.**			
Product	Company	Product Number	Final Concentration
B-estradiol (E2)	Sigma Aldrich	E4389	10 nM
Progesterone (P4)	Sigma Aldrich	P7556	1 μM
Dibutyryl cAMP sodium salt (db-cAMP)	Medchem Express	HY-B0764	0.5 μM

Supplementary Table S2. Composition of human endometrial organoid expansion medium (hEOM) and details of hormonal treatments used in the study.

**Supplementary Table S3: Oligonucleotide primer sequences used for RT-qPCR analysis.**	
Gene	Primer sequence (5'-3')
ESR1	F:TGGGAATGATGAAAGGTGGGAT
	R:GGTTGGCAGCTCTCATGTCT
PGR	F:CATGGTCCTTGGAGGTCGAAA
	R:TGTGGGAGAGCAACAGCATC

FOXO1	F:CTACGAGTGGATGGTCAAGAGC
	R:CCAGTTCCTTCATTCTGCACACG
HSD17B2	F:TCCAACCTGGAGGCTTCCTAAC
	R:GCTGTGCTAAGATGTAGTCCTGG
LIF	F:GTCTTGGCGGCAGTACACA
	R:ACGACTATGCGGTACAGCTC
MUC1	F:CCTACCATCCTATGAGCGAGTAC
	R:GCTGGGTTTGTGTAAGAGAGGC
PAEP	F:CTGGAGCTCCCAAAGGCCC
	R:CGATCTCCAGGTTGTCCTCGG
SPP1	F:CGAGGTGATAGTGTGGTTTATGG
	R:GCACCATTCAACTCCTCGCTTTC
GAPDH	F:GGTATCGTGGAAGGACTCATGAC
	R:ATGCCAGTGAGCTTCCCGTTCAG
HPRT1	F:CATTATGCTGAGGATTTGGAAAGG
	R:CTTGAGCACACAGAGGGCTACA

Supplementary Table S3. Oligonucleotide primer sequences used for RT-qPCR analysis.

**Supplementary Table S4: Details of antibodies and dilutions used in the study**			
Product	Company	Product number	Final concentration
Anti-ERα Antibody (MC-20)	SantaCruz Biotech	sc-542	1:100
Ki-67	Novus Biologicals	NB500-170	1:400
Progesterone Receptor A/B (D8Q2J)	Cell Signaling	#8757	1:200
E-Cad	Cell Signaling	9835S	1:200
PAEP	Atlas Antibodies	HPA029473	1:100
Cytokeratin	Abcam	ab86734	1:100
Vimentin	Abcam	Ab 92547	1:100
Alexa fluor 488	Invitrogen	A-11034	1:1000
Alexa fluor 568	Invitrogen	A-11004	1:1000

Supplementary table S4: Details of antibodies and dilutions used in the study
